# Diet and Consumer Behavior in U.S. Vegetarians: A National Health and Nutrition Examination Survey (NHANES) Data Report

**DOI:** 10.3390/ijerph19010067

**Published:** 2021-12-22

**Authors:** Maximilian Andreas Storz, Alexander Müller, Mauro Lombardo

**Affiliations:** 1 Department of Internal Medicine II, Center for Complementary Medicine, Freiburg University Hospital, Faculty of Medicine, University of Freiburg, 79106 Freiburg, Germany; alexander.mueller@uniklinik-freiburg.de; 2 Department of Human Sciences and Promotion of the Quality of Life, San Raffaele Roma Open University, 00166 Rome, Italy; mauro.lombardo@uniroma5.it

**Keywords:** vegetarian, plant-based diet, nutrition, consumer behavior, food security

## Abstract

An increasing number of individuals adopt plant-based diets for their potential health benefits. Understanding vegetarians’ dietary behavior in the context of their socioeconomic background is essential for pro-vegetarian messaging and to influence public beliefs about plant food consumption. Thus, this study sought to investigate diet and consumer behaviors in U.S. vegetarians. This is a cross-sectional, population-based study with data from the Nutrition and Health Examination Surveys (2007–2010). Selected items from three modules (diet and nutrition behavior, consumer behavior, and food security) were compared between vegetarians (*n* = 352) and the general population (*n* = 14,328). U.S. vegetarians consumed significantly fewer calories and less cholesterol but more fiber than their omnivorous counterparts. Moreover, vegetarians had significantly fewer soft drinks and salty snacks available at home. We also observed significant intergroup differences with regard to the availability of fruit and dark green vegetables. Vegetarians spent less money on eating out and indicated a lower number of not-home-prepared meals and ready-to-eat foods. We found no differences regarding money spent at supermarkets or grocery stores. Our study contributes to a better understanding of dietary and consumer behaviors in vegetarians. We shed a new light on the economic feasibility of vegetarian diets, highlighting that these diets are not necessarily more expensive than an omnivorous diet.

## 1. Introduction

Vegetarian and vegan plant-based diets have been associated with a myriad of health benefits, including improved body weight and postprandial metabolism [1], lower blood pressure levels [2], and protective effects vs. the incidence of ischemic heart disease and cancer [3].

In light of these benefits, more and more individuals have reduced their meat consumption and adopted a plant-based diet [4,5]. As such, attitudes toward vegetarian diets are gradually changing [6]. This ongoing trend has been substantially accelerated with the advent of COVID-19 [7,8] It is, however, not only for health reasons why individuals sympathize with a plant-based diet. Ethical, environmental, religious and, most importantly, socioeconomic aspects also play a crucial role in this process [9,10,11,12].

As a consequence, vegetarian populations around the globe substantially differ in size and in their dietary habits [13]. Understanding motivations, perceived benefits and barriers of consuming a plant-based diet will help formulate targeted pro-vegetarian messaging to influence beliefs about plant food consumption, and, ultimately, public health [14,15].

In recent years, research has used data from the National Health and Nutrition Examination Survey (NHANES) to characterize and describe vegetarians in the United States of America [16]. This population has been meticulously analyzed with regard to demographic and socioeconomic characteristics and with regard to food intake patterns [15,17].

Little is known, however, about diet and consumer behaviors as well as food security status in this population. The present study attempted to change this. We sought to investigate whether U.S. vegetarians differ significantly from the rest of the U.S. population with regard to the aforementioned aspects.

## 2. Materials and Methods

### 2.1. Study Design and Population

The main aim of this study was to investigate differences in diet and consumer behaviors between vegetarians and non-vegetarians. We used cross-sectional, population-based data from the Nutrition and Health Examination Surveys to investigate diet and consumer behavior in U.S. vegetarians and to contrast the results to U.S. individuals who did not have any special diet. The National Health and Nutrition Examination Survey is a representative survey research program designed to assess the health and nutritional status of the U.S. civilian non-institutionalized resident population [16]. The survey examines a nationally representative sample of about 5000 persons per year. One of its hallmarks is the complex multistage, stratified, clustered, probability sampling design [18]. Data are gathered via questionnaire-based personal interviews at participants’ homes, followed by visits at a mobile examination center [19].

For this particular study, we appended 2 consecutive NHANES survey cycles (2007–2008 and 2009–2010) that both included a “vegetarian status” variable. The study design is cross sectional and all NHANES survey protocols were approved by the NCHS Research Ethics Review Board (ERB) (Protocol Number: Protocol #2005-06).

### 2.2. Data Collection Instruments

The NHANES interview includes demographic, dietary, socioeconomic and other health-related questions [16]. The examination component includes medical and physiological measurements administered by highly trained medical personnel. For the purpose of this study, we merged demographic data, examination data, dietary data and questionnaire data.

Demographic data included age, gender, and ethnicity [20]. Race/ethnicity was divided into five categories: Mexican American, non-Hispanic White, non-Hispanic Black, other Hispanic, and Other Race (includes mixed race).

Dietary data included daily calorie intake as well as fiber intake, cholesterol intake, alcohol intake, and usage of ordinary salt or seasoned salt added in cooking or preparing foods [21]. We obtained estimates of energy intake using the first 24 h, interviewer-based, dietary recall from both survey cycles (2007–2008 and 2009–2010). The NHANES is one of the best sources of a national-level diet [22], and dietary recall interviews were conducted in person by trained dietary interviewers fluent in English and Spanish. Interviewers used a computer-assisted dietary interview software program (USDA’s automated multiple-pass method by ARS Food Surveys Research Group (FSRG), Beltsville, MD, USA). Participants were asked to describe all foods and beverages consumed on the preceding day (between midnight and midnight) as part of the dietary interview. The exact methods have been described in great detail by Malek and colleagues in one of their recent publications [22].

Previous analyses of nutrient intake in NHANES vegetarians (2007–2010) have already revealed that these individuals consume, on average, significantly fewer calories than their omnivorous counterparts [17]. Thus, we employed a commonly used energy adjustment method to account for this phenomenon and expressed nutrient density as intake (in gram or milligram)/1000 kcal. Adjustment for total energy intake is an appropriate tool in epidemiological studies to control for confounding, and to reduce extraneous variation [23,24].

Examination data were limited to anthropometric measurements including body weight, body height and body mass index (BMI) [25]. The body measurement data were collected by trained health technicians. A detailed procedure manual was posted on the NHANES 2007–2008 website [26].

The major focus of this study lay on questionnaire data and included three major sections: (a) consumer behavior, (b) diet and nutrition behavior, and (c) food security [27].

The consumer behavior module focused on a core set of questions asked during the household interview and investigated two major topics: the availability of certain types of foods in the family and family food expenditures in general. Participants were asked how often they had a certain type of food at home (see below). Potential replies included “always”, “most of the time”, “sometimes”, “rarely”, or “never”. Foods included fruits (including fresh, dried, canned and frozen fruits), dark green vegetables (including fresh, dried, canned, and frozen vegetables), salty snacks (chips and crackers), and soft drinks (including fruit-flavored drinks, or fruit punch).

Food expenditures questions inquired about the amount of money spent on foods in the past 30 days. Initially, information was collected using two-part questions (number and unit) to allow respondents to report the amount of money as either per week or per month. Afterwards, replies were standardized. Participants were asked about the amount of money spent on food at supermarkets or grocery stores (including food stamp purchases) and the amount of money spent on food at other stores. Furthermore, interviewers asked participants about the amount of money spent on eating out (e.g., at cafeterias, at work or on vending machines) and spent on carryout/delivered foods. An additional question targeted at the frequency of major food shopping. In light of the high number of missing values, we intentionally refrained from including the consumer behavior phone follow-up module (see below).

The diet behavior and nutrition module investigated individual participants’ food choices. Participants were asked about the number of non-home-prepared meals (e.g., meals obtained from places such as restaurants, fast food restaurants, food stands, grocery stores, or from vending machines) over the previous 7 days. A follow-up question inquired about the number of ready-to-eat foods consumed in the past 30 days. This question included salads, soups, chicken, sandwiches and cooked vegetables from salad bars and deli counters. Ultimately, individuals were asked about the number of frozen meals or frozen pizzas consumed in past 30 days.

The diet behavior and nutrition module also inquired as to whether participants perceived themselves as vegetarians or not. As such, the assessment of vegetarian status was not based on nutritional protocols but on a subjective self-evaluation. Only individuals providing a definite answer (yes or no) to this question were included for the present study.

Finally, our analysis included questions from the food security module. The 3 questions from the household food security section included: “(I/we) worried whether (my/our) food would run out before (I/we) got money to buy more”, “The food that (I/we) bought just didn’t last, and (I/we) didn’t have money to get more”, and “(I/we) couldn’t afford to eat balanced meals. Response categories included “often true”, “sometimes true”, and “never true”.

### 2.3. Statistical Analysis

We used STATA 14 statistical software (StataCorp. 2015. Stata Statistical Software: Release 14. StataCorp LP, College Station, TX, USA) for data analysis. The “svyset” and “svy” commands were used to account for the complex NHANES survey design characteristics and the population weights. For this analysis, we generated a 4-year-weight (2007–2010) for the interview data. To preserve the main survey design and to provide larger standard errors, we performed unconditional subclass analyses to compare the aforementioned variables between non-vegetarians and vegetarians [28].

Normally distributed variables were described with their mean and standard error in parentheses (see below). Standard errors were computed using procedures that took into account the complex nature of the NHANES sample design. This is a common practice when analyzing complex survey data and allows for statistically valid population inferences from sample data [29].

Comparisons were made between self-identified vegetarians and non-vegetarians (denying any other special diet) using appropriate parametric tests where data were normally distributed. An assessment of a special diet was performed using the NHANES dietary interview question “are you currently on any kind of diet, either to lose weight or for some other health-related reason?” (variable “on a special diet”). Individuals who answered “yes” were excluded from the non-vegetarian group.

We used STATA’s post-estimation command “lincom” to make the aforementioned comparisons between both subpopulations. All tests were two-tailed and a *p*-value of 0.05 was used for statistical significance. Pie charts were used to display the results from the food security questions over both groups. Only individuals with a full dataset (without missing information on any of the aforementioned variables) were included in the final analysis.

## 3. Results

**Figure 1:**
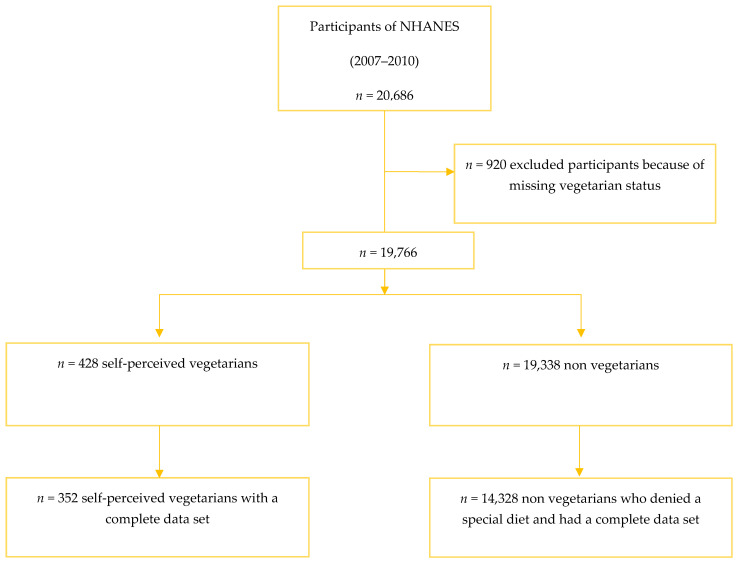
Inclusion flow diagram.

We included *n* = 14,680 individuals that had a full dataset in our analysis. We identified *n* = 352 (self-perceived) vegetarians and *n*= 14,328 non-vegetarians that indicated they were not on any kind of special diet (Figure 1). A total of 920 individuals (488 for the 2007/2008 cycle and 432 for the 2009/2010 cycle, respectively) were excluded from the analysis because of a missing answer to the question “do you consider yourself to be a vegetarian?”.

Table 1 summarizes selected demographic and anthropometric aspects of the included individuals. With regard to age, we observed no significant differences between both groups. The proportion of females, however, was significantly higher in the vegetarian group as compared to the control group (*p* < 0.001). Vegetarians also exhibited a significantly more favorable BMI than non-vegetarians (*p* < 0.001). The proportion of Mexican American and Non-Hispanic Black vegetarians was significantly smaller than in the general population (*p* = 0.049 and 0.002, respectively).

Daily calorie intake (kcal/d) and the consumption of selected nutrients (energyadjusted, units per 1000 kcal) is shown in Table 2. Non-vegetarians consumed significantly more calories (*p* = 0.002) and cholesterol (*p* < 0.001) than vegetarians, whereas vegetarians consumed significantly more fiber (*p* = 0.001). We observed no statistically significant differences in alcohol intake.

Table 3 summarizes diet behavior (top) and consumer behavior (bottom) by vegetarian status. The proportion of non-vegetarians “never” having fruits and dark green vegetables at home was significantly larger as compared to vegetarian individuals (*p* ≤ 0.01). The same applied for the category “rarely” for the vegetable item. In contrast, the proportion of non-vegetarians having fruits available “most of the time” was significantly higher (*p* = 0.031). Apart from this, we observed no additional significant intergroup differences in proportions in the frequency of fruits and dark green vegetables availability at home (*p* > 0.05 for all).

The proportion of vegetarians “always” having salty snacks available at home was significantly lower as compared to non-vegetarians (34.67% vs. 43.65%, *p* = 0.008). Approximately 6.1% of vegetarians stated that they “never” had salty snacks at home, whereas this was true for only 2.98% of non-vegetarians (*p* = 0.021).

A comparable picture was observed with regard to the availability of soft drinks (Table 3). The proportion of individuals “always” having soft drinks available at home was significantly higher in non-vegetarians (41.89%) as compared to vegetarians (21.06%). Almost 23% of vegetarians indicated that they “never” had soft drinks at home; a statement that applied for only 12.83% of non-vegetarians. Comparable significant differences were found with regard to those “rarely” having soft drinks at home.

The proportion of vegetarians “never” or “rarely” using ordinary salt or seasoned salt added in cooking or preparing foods tended to be higher as compared to non-vegetarians (Table 3); however, the results failed to reach statistical significance.

Non-vegetarians spent significantly more money on eating out than vegetarians (USD 158.96 vs. USD 126.08; *p* = 0.005). The amount of money spent on carryout and delivered foods was almost equal in both groups. We found no significant intergroup differences with regard to money spent on food at supermarket/grocery stores and money spent on food at other stores. There was no difference in the frequency of major food shopping between both groups.

Of note, non-vegetarians ate significantly more meals that were not home prepared in the past seven days (3.55 vs. 2.46, *p* < 0.001). However, we found no significant differences with regard to the number of frozen meals consumed within the past 30 days. The total number of ready-to-eat foods consumed in the past 30 days was significantly lower in the vegetarian group (see Table 3).

**Figure 2:**
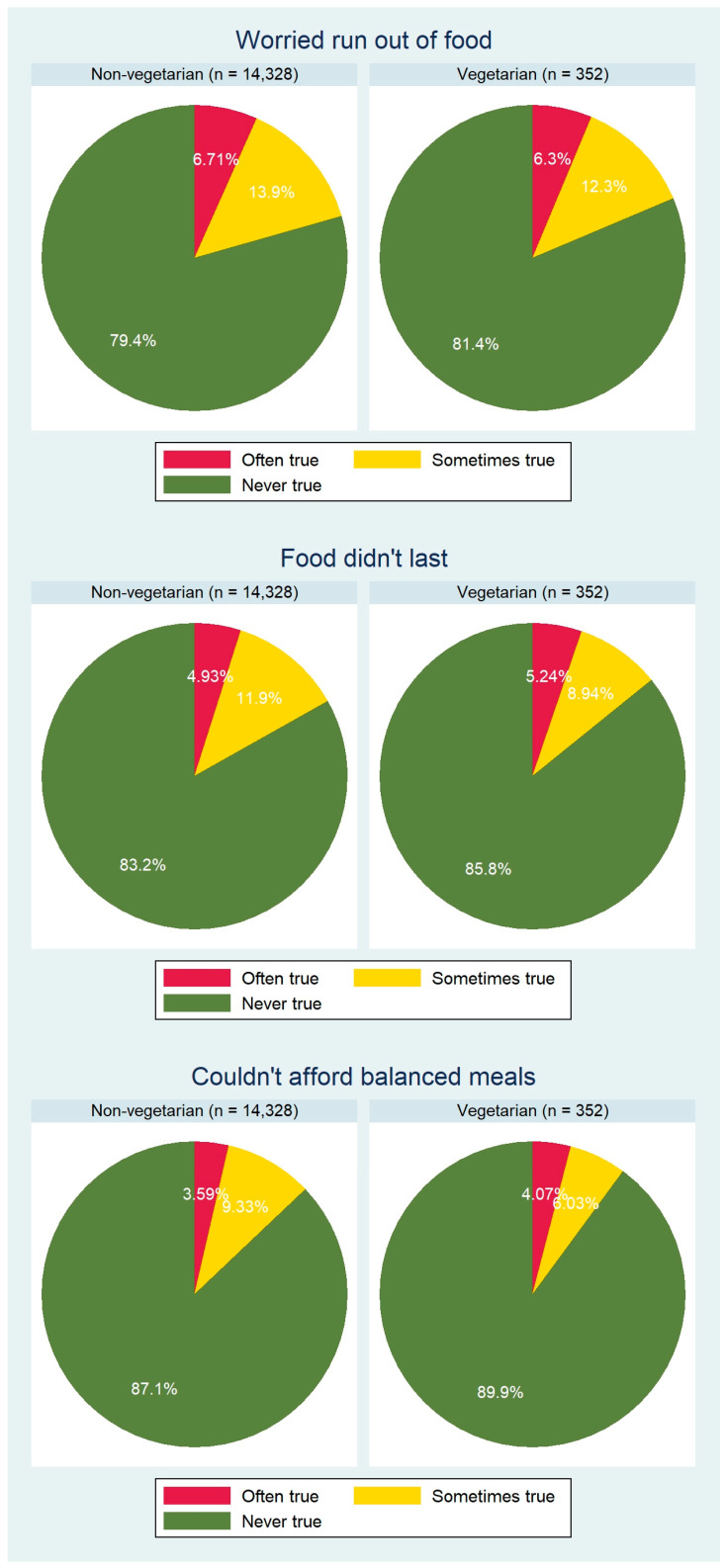
Food security analysis in non-vegetarians vs. vegetarians: an overview.

Figure 2 shows the results of the three selected questions that investigated food security level in both groups at the household level. We found no significant intergroup differences in proportions with regard to the first two items (*p* > 0.05 for both questions). Of note, the proportion of non-vegetarians that sometimes could not afford balanced meals was significantly higher than in vegetarians (9.33% vs. 6.03%; *p* = 0.026).

## 4. Discussion

This study investigated dietary and consumer behavior in U.S. vegetarians and compared the results to the general population that denied consuming any special diet. Our results confirmed the initial hypothesis that significant differences exist between non-vegetarians and vegetarians. Compared to their omnivorous counterparts, U.S. vegetarians consumed significantly less calories and cholesterol and significantly higher amounts of fiber (Table 2). Vegetarians had significantly fewer soft drinks and salty snacks at home. The proportion of non-vegetarians “never” having fruits and dark green vegetables at home was significantly larger as compared to vegetarian individuals (*p* ≤ 0.01, Table 3). Finally, vegetarians spent less money on eating out and also indicated a lower number of not-home-prepared meals and ready-to-eat foods (Table 3).

Our findings are somewhat surprising and yet expected at the same time. In general, individuals consuming a (vegetarian or vegan) plant-based diet often exhibit a healthier lifestyle (as compared to omnivores) [30,31]. For example, vegetarians are more likely to be non-smokers [32] and are more physically active compared to non-vegetarians [33]. In our study, vegetarians spent less money on eating out and indicated a lower number of not-home-prepared meals.

In a 2017 study, eating home-cooked meals more frequently was associated with better dietary quality and lower adiposity levels [34]. As such, our findings align well with the healthier lifestyle of vegetarians and could be interpreted as an indicator that vegetarians pay more attention to what (and where) they eat. We believe this to be important in light of the fact that US adults have decreased their consumption of foods from their home supplies and reduced time spent cooking since 1965 [35].

In our analysis, vegetarians also spent significantly less money on eating out (see Table 3). Of note, the proportion of non-vegetarians who were sometimes unable to afford balanced meals was significantly higher than in vegetarians (9.33% vs. 6.03%; *p* = 0.026). Our additional food security question analysis indicated no significant intergroup differences (Figure 2), which could be interpreted in the way that not eating out is not a financial matter but a health-conscious choice. We believe this to be an indicator that vegetarians in our sample are not “vegetarians of necessity” (people for whom meat is not readily available, e.g., because it is too expensive) but “vegetarians of choice”. The point must be made, however, that the cross-sectional nature of our data does not allow for causal attributions.

Our findings are in accordance with the aforementioned study by Juan and colleagues [17]. Juan et al. found that U.S. vegetarians consumed significantly more fruits, vegetables, whole grains, and total grains than non-vegetarians (after energy adjustment, per 1000 kcal). Our food item availability analysis revealed that the proportion of individuals always having dark green vegetables at home is, in fact, higher than in non-vegetarians (Table 3); however, the results failed to reach statistical significance. In addition, the proportion of non-vegetarians “never” having fruits and dark green vegetables at home was significantly larger as compared to vegetarian individuals (*p* ≤ 0.01). With regard to the study by Juan and colleagues, one could have expected a larger (and statistically significant) intergroup difference in our sample.

Both recall bias and social acceptability bias may be potential explanations for this observed phenomenon. Yet, in light of the cross-sectional nature of our data, we may only speculate about this. After all, it is noteworthy that fruit and vegetable availability is higher in vegetarians, and particularly the consumption of vegetables has been association with improved health outcomes [36].

In contrast, we found no significant differences with regard to the number of frozen meals between both groups. Ready-made meals were found to be associated with a higher energy intake and may increase the risk of central obesity and fat deposition [37]. In light of the more favorable body weight in vegetarians in this sample, one could have expected a lower number of frozen meals in this group.

Finally, financial aspects warrant additional investigation. As mentioned before, vegetarians in our study are probably not “vegetarians of necessity” who cannot afford meat and meat products. Both groups spent equal amounts at supermarkets and grocery stores (Table 3). The same applies for carryout and delivered foods. With regard to the food security questions, we found a significantly higher proportion of non-vegetarians that sometimes could not afford balanced meals (Figure 2). With regard to the other items, we found no significant intergroup differences. A frequently encountered argument against the vegetarian or vegan diets is the high price of fruits and vegetables. Interestingly, an older U.S. study indicated that consumers can meet the recommendation of three servings of fruits and four servings of vegetables daily for 64 cents [38]. One must take into account that food prices increased significantly over the past two decades, particularly for fresh fruits and vegetables. Eight years ago, it was possible for an individual on a 2000-calorie diet to eat a sufficient quantity and variety of fruits and vegetables for about USD 2.10 to USD 2.60 per day [39]. Whether this is still possible today, is, to the best of our knowledge, unknown, and newer studies are urgently required.

Nevertheless, it should be clear that governments, public health authorities and other involved stakeholders should make every attempt possible to ensure that people are able of meeting the recommended daily food and vegetable intake. This is also important from an environmental health point of view, as industrial meat as well as animal food production exert a huge negative influence over the environment [40] and is unsustainable [41]. Long-term strategies to initiate sustainable and long-lasting food choices are therefore urgently warranted [42].

The fact that both compared groups in our trial spent approximately the same amount of money on shopping at grocery stores and supermarkets highlights that a vegetarian diet is not a “luxury” for privileged people but could theoretically be adopted by the average citizen as well. A study by Lusk and Norwood confirmed this trend and found that “true” vegetarians do indeed report lower food expenditures as compared to omnivores [43].

The aim of this study was to investigate diet and consumer behavior as well as food security in the U.S. vegetarian population. Our results confirm the hypothesis that U.S. vegetarians differ significantly from the rest of the U.S. population with regard to the aforementioned aspects, and appear, overall, to have healthier diets. Our findings are of paramount importance in the discussion about the economic feasibility of vegetarian diets. While our results allow new insights into this topical subject, several limitations need to be discussed.

### Limitations

Our study has several strengths and limitations that warrant further discussion. The main weakness is probably that the current status of the vegetarian diet was self-reported. Using the same set of NHANES data, Juan et al. demonstrated that several vegetarians in this sample also reported the consumption of some types of animal products, such as meat or seafood [17]. In contrast, the percentage of vegans in this sample is rather low. As such, a high level of caution is warranted when interpreting the term “vegetarian” from self-reports. A Finnish study revealed that many self-identified vegetarians did not follow a vegetarian diet according to frequently operationalized definitions [44]. Although many self-identified vegetarians occasionally consume meat and fish, they eat a healthier diet than self-defined omnivores. These factors should be considered when interpreting the results of the present study.

Another weakness is that, unlike initially planned, we were not able of including the NHANES “consumer behavior phone follow-up module” in our study. Adding this subset of parameters would have further decreased the number of eligible cases to *n* = 208 with a complete dataset, and, as such, we refrained from this step.

With *n* = 352 cases, the number of included vegetarians is already relatively small and we acknowledge that the percentage of vegetarians in the U.S. general population grew significantly since 2010. The data we used for this analysis date back to 2010, and a lot of things may have changed in the field of vegetarian nutrition over the last decade. It is not inconceivable that the growing availability of plant-based meat and cheese alternatives (as well as the increasing number of vegetarian and vegan restaurants) may have altered vegetarians’ dietary and consumer behaviors. On the other hand, one must consider that newer NHANES cycles no longer contain the vegetarian status variable. As such, it was impossible for us to add additional (newer) survey cycles.

Finally, our data are cross sectional and no causal inference can be drawn from this type of dataset. The associations identified are often more difficult to interpret, as compared to well-designed randomized-controlled trials with a clear endpoint. For the descriptive nature of our paper, however, we believe that NHANES data may be an adequate choice.

As for the strengths, we present a large and nationally representative dataset (National Health and Nutrition Examination Survey) in a field (dietary and consumer behavior in vegetarians) that is largely unexplored. To the best of our knowledge, this is one of the first studies to investigate consumer behavior in U.S. vegetarians using NHANES data.

## 5. Conclusions

The present study added to a better understanding of dietary and consumer behavior in U.S. vegetarians. A lower number of not-home-prepared meals and a lower home availability of salted snacks and soft drinks characterized the vegetarian sample in this study. The key finding that vegetarians and omnivores spent equal amounts of money in grocery stores and supermarkets is of high relevance in the discussion about the economic feasibility of a vegetarian diet. Future studies should elucidate potential economic barriers to the adoption of plant-based diets based on larger and more current data.

## Figures and Tables

**Table 1:** Demographic and anthropometric characteristics by (self-perceived) vegetarian status: a comparison.

	Non-Vegetarians *n* = 14,328	Vegetarians *n* = 352	*p*-Value
Sex			
Female	*n* = 7044 (49.16%)	*n* = 237 (67.34)	<0.001
Male	*n* = 7284 (50.84%)	*n* = 115 (32.66%)	<0.001
Ethnicity			
Mexican American	*n* = 1461 (10.20%)	*n* = 26 (7.40%)	0.049
Other Hispanic	*n* = 759 (5.3%)	*n* = 20 (5.82%)	0.594
Non-Hispanic White	*n* = 9537 (66.56%)	*n* = 198 (56.17%)	0.071
Non-Hispanic Black	*n* = 1759 (12.28%)	*n* = 27 (7.59%)	0.004
Other Race-Including Multi-Racial	*n* = 812 (5.67%)	*n* = 81 (23.02%)	0.004
Age (years)	36.73 (0.39)	36.27 (1.55)	0.767
Weight (kg)	71.34 (0.39)	63.49 (1.39)	<0.001
Height (cm)	161.70 (0.33)	158.79 (1.21)	0.021
BMI (kg/m²)	26.12 (0.08)	24.19 (0.39)	<0.001

Values for continuous variables are expressed as estimated mean and standard error in parenthesis. A *p*-value < 0.05 indicates significant differences in the proportions.

**Table 2:** Intake of calories (kcal/d) and selected nutrients (energy-adjusted) by vegetarian status: a comparison.

	Non-Vegetarians *n* = 14,328	Vegetarians *n* = 352	*p*-Value
Calories (kcal/d)	2143.09 (12.91)	1901.52 (71.51)	0.002
Fiber (gm/1000 kcal)	7.56 (0.10)	11.11 (0.40)	0.001
Cholesterol (mg/1000 kcal)	129.84 (1.44)	85.25 (6.49)	<0.001
Alcohol (gm/1000 kcal)	3.52 (0.16)	2.45 (0.58)	0.071

Values are expressed as estimated mean and standard error in parenthesis. A *p*-value < 0.05 indicates a significant intergroup difference.

**Table 3:** Diet and consumer behavior by (self-perceived) vegetarian status: a comparison.

	Non-Vegetarians *n* = 14,328	Vegetarians *n* = 352	*p*-Value
**Dietary Behavior**			
Salt usage			
Never	*n* = 977 (6.82%)	*n* = 35 (9.92%)	0.074
Rarely	*n* = 2721 (18.99%)	*n* = 65 (18.432%)	0.863
Occasionally	*n* = 5202 (36.31%)	*n* = 122 (34.73%)	0.647
Very Often	*n* = 5266 (36.75%)	*n* = 126 (35.89%)	0.844
Don’t know	*n* = 162 (1.13%)	*n* = 4 (1.03%)	0.828
Fruits available			
Always	*n* = 9742 (67.99%)	*n* = 263 (74.94%)	0.113
Most of the time	*n* = 2709 (18.91%)	*n* = 46 (13.15%)	0.031
Sometimes	*n* = 1407 (9.82%)	*n* = 33 (9.33%)	0.878
Rarely	*n* = 397 (2.77%)	*n* = 9 (2.47%)	0.831
Never	*n* = 73 (0.51%)	*n* = 1 (0.11%)	0.01
Dark green vegetables available			
Always	*n* = 7853 (54.81%)	*n* = 218 (61.90%)	0.057
Most of the time	*n* = 3291 (22.97%)	*n* = 84 (23.90%)	0.738
Sometimes	*n* = 2210 (15.42%)	*n* = 42 (11.88%)	0.115
Rarely	*n* = 638 (4.45%)	*n* = 6 (1.82%)	0.03
Never	*n* = 336 (2.35%)	*n* = 2 (0.50%)	0.002
Salty snacks available			
Always	*n* = 6254 (43.65%)	*n* = 122 (34.67%)	0.008
Most of the time	*n* = 2917 (20.36%)	*n* = 60 (16.97%)	0.272
Sometimes	*n* = 3310 (23.10%)	*n* = 95 (26.92%)	0.217
Rarely	*n* = 1420 (9.91%)	*n* = 54 (15.32%)	0.145
Never	*n* = 427 (2.98%)	*n* = 22 (6.12%)	0.021
Soft drink available			
Always	*n* = 6002 (41.89%)	*n* = 74 (21.06%)	<0.001
Most of the time	*n* = 2068 (14.43%)	*n* = 56 (16.01%)	0.622
Sometimes	*n* = 2404 (16.78%)	*n* = 57 (16.18%)	0.832
Rarely	*n* = 2016 (14.07%)	*n* = 85 (24.06%)	0.026
Never	*n* = 1838 (12.83%)	*n* = 80 (22.69%)	0.02
**Consumer Behavior**			
Money spent at supermarket/grocery store ($)	450.45 (19.81)	475.97 (24.93)	0.409
Money spent on food at other stores ($)	65.24 (2.85)	63.48 (6.74)	0.803
Money spent on eating out ($)	158.96 (5.44)	126.08 (9.11)	0.005
Money spent on carryout/delivered foods ($)	25.14 (1.38)	25.99 (2.57)	0.717
Frequency of major food shopping			
More than once a week	*n* = 1908 (13.32%)	*n* = 57 (16.18%)	0.337
Once a week	*n* = 6827 (47.65%)	*n* = 164 (46.51%)	0.755
Once every two weeks	*n* = 3704 (25.85%)	*n* = 94 (26.79%)	0.743
Once a month or less	*n* = 1639 (11.44%)	*n* = 33 (9.40%)	0.225
Rarely make any major shopping trips, only small trips	*n* = 181 (1.26%)	*n* = 3 (0.86%)	0.541
Rarely shop for food	*n* = 69 (0.48%)	*n* = 1 (0.26%)	0.4
# of times someone cooked dinner at home	5.55 (0.40)	5.42 (0.15)	0.748
# of meals not home prepared	3.55 (0.06)	2.46 (0.21)	<0.001
# of ready-to-eat foods in past 30 days	1.68 (0.05)	1.16 (0.20)	0.01
# of frozen meals/pizza in past 30 days	2.90 (0.11)	2.48 (0.40)	0.341

Values for continuous variables are expressed as estimated mean and standard error in parenthesis. A *p*-value < 0.05 indicates significant differences in the proportions.

## Data Availability

Data are publicly available online (https://wwwn.cdc.gov/nchs/nhanes/Default.aspx; accessed on 19 December 2021). The datasets used and analyzed during the current study are available from the corresponding author on reasonable request.
